# Do antibiotic-impregnated shunts in hydrocephalus therapy reduce the risk of infection? An observational study in 258 patients

**DOI:** 10.1186/1471-2334-7-38

**Published:** 2007-05-08

**Authors:** Rainer Ritz, Florian Roser, Matthias Morgalla, Klaus Dietz, Marcos Tatagiba, Bernd E Will

**Affiliations:** 1 Department of Neurosurgery, University Hospital Tübingen, Hoppe-Seyler-Str. 3; 72076 Tübingen, Germany; 2 Department of Medical Biometry, University of Tübingen, Westbahnhofstr. 55; 72070 Tübingen, Germany

## Abstract

**Background:**

Shunt infection in hydrocephalus patients is a severe, even life-threatening complication. Antibiotic-impregnated shunts (AIS) have been developed in an attempt to reduce rate of shunt infection. The study was performed to analyze if AIS can diminish the rate of shunt infection. The pathogenic nature of shunt infection in patients with AIS systems and those without antibiotic impregnated shunts (non-AIS) was compared.

**Methods:**

Over a period of 24 months in the Department of Neurosurgery at University Hospital of Tübingen shunt surgery was performed in 258 patients. In 86 patients AIS systems were implanted. Shunt catheters were commercially impregnated with clindamycin and rifampicin. Analysis of the clinical data included sex, age, classification of hydrocephalus, shunt types and risk factors for shunt infection [age (< 1 year and > 80 years), prematurely born patients, external ventricular drainage, former shunt infection, former systemic infection, disturbance of consciousness, former radiation-/chemotherapy]. Infection rates and underlying bacterial pathogens of patients with AIS were compared to patients with implanted non-AIS systems (172 patients).

**Results:**

AIS and non-AIS patients did not differ in sex, etiology of hydrocephalus and the shunt type. In the AIS group 72 out of 86 patients had at least one risk factor (83.7 %), compared to 126 patients in the non-AIS group (73.3 %). There was no significant difference between the two groups (p = 0.0629; Fisher's exact test). In patients with no risk factors, only one patient with non-AIS suffered from shunt infection. In patients with one or more risk factors the rate for shunt infection was 7.14 % in patients with non-AIS and 6.94 % in patients with AIS. Former shunt infection (p = 0.0124) was related to higher risk for shunt infection. The use of AIS had therefore no significant advantage (p = 0.8611; multiple logistic regression).

Significantly related to a shunt infection was the number of shunt surgeries. 190 interventions in the AIS group (2.21 interventions per patient) and 408 in the non-AIS group (2.37 interventions per patient) had been performed (p = 0.3063; Wilcoxon). There was no shunt infection in the group of patients on whom only one shunt surgery was performed. In patients with at least two shunt surgeries the infection rate was 9%. The infection rate in AIS patients was 5/52 (9.6 %) and in the non-AIS 10/114 (8.77 %), (p = 1.0; Fisher's exact test). Staphylococcus epidermidis was the most frequent pathogen for shunt infection. Fourteen out of 15 infections occurred within the first 6 months of surgery. The most frequent pathogen for shunt infection was S. epidermidis. No toxic or allergic complications were seen using the AIS shunt systems. The presented data show a remarkably low infection rate of 5.8 % in the non-AIS group compared to other studies which demonstrated a significant decrease in the infection rate by AIS.

**Conclusion:**

AIS did not significantly reduce shunt infection in hydrocephalus patients in the presented study. In the AIS group three patients suffered from shunt infections caused by skin ulceration or neurosurgical procedures with exposure of the cerebrospinal liquor after shunt implantation. AIS was not developed to prevent infection in such cases, therefore an advantage of AIS can not be excluded. In view of the presented data and the small number of reported studies a prospective randomized multicenter study is required.

## Background

Shunt infection is a serious complication causing persisting intellectual, cognitive and neurological deficits [1]. It may be even a life threatening complication. In addition to this, severe individual impairment cost of complication management sums up to 27,000 to 30,000 $ per patient [2].

The rate of shunt infection varies from 0.3 to 12.9 % [2-9]. Risk factors for shunt infection include surgeon's experience, duration of surgery, and intraoperative handling of the device. Further unfavorable conditions for shunt operations are late timing of surgery and overcrowded operating theatres [10,11]. These factors can be influenced by rigorous planning and improved organisation [12]. Already in 1992, Choux et al. [13] established rules for the operating theatre to reduce shunt infection by influencing these risk factors. They were able to reduce their infection rate in 600 children to 0.33 % by strictly complying with the so-called „Choux's rules“. In 2005, Choksey and Malik reported only one shunt infection in a total of 176 shunt procedures performed in 126 patients with hydrocephalus due to strictly adhering to the following surgical principles: asepsis, antisepsis, antimicrobial therapy, and the avoidance of haematomas [11]. In contrast, several risk factors causing shunt infection cannot be influenced: patients' age, suppressed immune system e.g. in patients receiving radiation and/or chemotherapy, in long-term intensive care, with a disturbed level of consciousness, skin alterations, previous infection and external cerebrospinal fluid drainage previous to shunt implantation.

A promising alternative is to use antibiotic-impregnated shunts (AIS) in order to reduce the rate of shunt infections.

The aim of the present study was to investigate whether AIS can diminish the rate of shunt infections. Risk factors for shunt infection were analysed. Furthermore, a comparison was made between the pathogenic nature of a shunt infection in patients with AIS systems and those without AIS (non-AIS).

## Methods

### Patients

Over a two-year period, 258 patients underwent shunt surgery in the Department of Neurosurgery at the University of Tübingen, Germany. Shunt insertions were performed by variably experienced surgeons (consultants and residents). The decision whether to implant an AIS or non-AIS system was made by the responsible surgeon. Shunt-catheters impregnated with clindamycin and rifampicin (Bactiseal, Codman, Johnson & Johnson, Boston, USA) were available for all patients.

#### Operative technique

With induction of general anesthesia, a single shot of 3 gram cefuroxim was given to adults intravenously. Children received 100 mg per kg body weight. Scalp hair was shaved widely before the skin was prepared with benzine. After marking skin incisions, the head, trunk and abdomen were prepared with alcoholic disinfection. The pinna of the ear was carefully taped forward. The operative area was draped, using sterile adhesive drapes, followed by a second layer of towels to cover the entire operating theatre table down to the floor level. The operative field was sealed by transparent sterile adhesive foil. Initially, the distal end of the abdominal catheter was implanted intraperitoneally through a transverse right incision paramedially to the umbilicus. The catheter was then tunneled up to the retroauricular area. A skin incision was performed 3 cm behind the ear to insert a programmable shunt (Hakim Programmable Valve Shunt, Codman, Johnson & Johnson, Boston, USA) with a tunneller. The final step was the insertion of the ventricular catheter. A paramedian right precoronary frontal burr hole was used whenever possible followed by opening the dura and diathermizing the arachnoid for the implantation of the ventricular catheter. The wounds were closed using 3-0 atraumatic sutures, then disinfected using betaisodona solution and finally covered with sterile adhesive plaster.

#### Definition of shunt infection

Shunt infection was defined when cerebrospinal fluid or the shunt tip was contaminated with bacteria and the patient showed clinical signs of acute bacterial meningitis and symptoms of shunt malfunction or obstruction. Additionally, one of the following parameters of bacterial inflammation of the cerebrospinal fluid (CSF) had to be positive: (i) leukocyte count of >0.25 × 10^9^/L with predominantly poly-morphonuclear cells, (ii) a CSF lactate concentration of >3.5 mmol/L, (iii) a glucose ratio (CSF glucose/serum glucose) of <0.4, (iv) a CSF glucose value of <2.5 mmol/L when no simultaneous value was noted in the blood glucose [14,15]. The antibiograms were specified by agar diffusion test according to the standards of NCCLS [16], alternatively the pathogens were tested by Vitek 2 [17].

### Ethical aspects

This study was approved by the ethical committee of the faculty of medicine of the Eberhard-Karls-Universität Tübingen.

### Statistical analysis

Dichotomous data were compared with Fisher's exact test. Continuous data were compared with the two-sample t-test. A Wilcoxon test was used to compare the two treatment groups with respect to the number of surgeries. Nominal data (classification of hydrocephalus) were compared by contingency table analysis. Multiple logistic regressions were used to investigate the influence of nominal factors together with the influence of treatment on the risk of a shunt infection. We first used a full model, which includes the interaction term. Since this turned out to be nonsignificant for all risk factors, the model was simplified to contain only the two main factors. For each test we calculated the corresponding p-value. All statistical methods were carried out with the statistical software package, JMP version 6.0.3 [18].

For determining the sample size required in a prospective study the software PS: Power and Sample Size Calculation, version 2.1.30 was used [19].

## Results

In 258 patients shunt procedures were performed in the Department of Neurosurgery, University of Tübingen, over a period of 24 months. AIS were used (Codman Bactiseal, Antimicrobial Impregnated catheter system) in 86 patients. The shunts were impregnated with clindamycin and rifampicin. Non-AIS were used in 172 patients.

Table 1 shows details comparing the two groups with respect to their age, sex, etiology of hydrocephalus, and the shunt type. The comparison of the AIS and non-AIS group showed no difference between the two groups in sex, etiology of hydrocephalus and the shunt type. The AIS group was about ten years younger than the non-AIS group. This is due to the fact that there were nearly twice as many children less than ten years in the AIS group compared to the non-AIS group. In Table 2, factors assumed to influence the risk for shunt infection are listed. In the AIS group, 72 out of 86 patients had at least one risk factor (83.7 %). In the non-AIS group 126 patients had at least one risk factor (73.3 %), i.e. we could not detect a significant difference between the two groups (p = 0.0629; Fisher's exact test). The risk of shunt infection by age is characterized in more detail in Table 3. Patients initially assumed for higher risk had an infection rate of 3 % compared to patients assumed for normal risk. Former shunt infection resulted in 18.5 % a shunt infection, whereas only 4.3 % with no former shunt infection suffered from a shunt infection (p = 0.0124 Fisher's exact test; OR 5.0; 95% CI 1.6 – 16.0) (Table 4).

**Table 1:** Demographic data, classification of hydrocephalus and shunt type

	**AIS **n = 86	**non-AIS **n = 172	P value
Mean age [years]	32.8	42.4	0.0071*^1^
Sex [female/male]	49/37	95/77	0.8943^2^
***Functional classification of hydrocephalus***			0.0971^2^
Obstructive /	17	50	
communicating /	68	114	
Not-classified	1	8	
***Shunt type***			0.3372^3^
Codman programmable	73	143	
Hakim	11	17	
Medos for children	1	2	
Others	1	10	

AIS patients were significantly younger than non-AIS patients.

^1^t-test. ^2^Fisher's exact test. ^3^Pearson. P significant at < 0.05.

**Table 2:** Risk factors for shunt infection independent of the treatment (AIS vs non-AIS)

**Risk factor**	**Odds Ratios**	**P value**
Age (< 1 year and > 80 years)	0.5 (0.06–3.7)	0.7010
Premature birth	2.1 (0.6–7.9)	0.2300
External ventricular drainage	1.9 (0.7–5.5)	0.2732
Former shunt infection	5.0 (1.6–16.0)	0.0124*
Former systemic infection	2.9 (1–8.8)	0.0605
Disturbance of consciousness	2.1 (0.6–7.9)	0.2300
Former Radiation-/Chemotherapy	2.2 (0.6–8.3)	0.2138

Odds ratio (OR) and 95 % confidence interval were calculated on the basis of Fisher's exact test, *significant at p < 0.05;

**Table 3:** Risk of shuntinfection by age

	no shuntinfection	shuntinfection	
Age [years] 1 – 80	211 (93.78 %)	14 (6.22 %)	225
Age [years] < 1 or > 80	32 (96.97 %)	1 (3.03 %)	33
	**243**	**15**	**258**

**Table 4:** Risk of shunt infection by former shuntinfection

	No shuntinfection	shuntinfection	
No former shuntinfection	221 (95.67 %)	10 (4.33 %)	231
Former shuntinfection	22 (81.48 %)	5 (18.52 %)	27
	**243**	**15**	**258**

The rates of shunt infections related to the risk factors and the kind of treatment (AIS vs non-AIS) are summarized in Table 5. In patients with no risk factors at all, only one patient with non-AIS suffered from shunt infection. In patients with one or more risk factors, the rate for shunt infection was 7.14 % in patients with non-AIS and 6.94 % in patients with AIS. If one disregards treatment the difference of 1.7% shunt infections in patients without risk factors and 7.1% in patients with at least one risk factor is not significant (p = 0.2038, OR = 4.5; 95% CI 0.6 – 34.9).

**Table 5:** Comparing rate of shuntinfection by risk factors and AIS vs non-AIS

Risk factors		No shuntinfection	Shuntinfection	n
No	non-AIS	45 (97.83%)	1 (2.17 %)	46
No	AIS	14 (100 %)	0 (0 %)	14
Yes	non-AIS	117 (92.86 %)	9 (7.14 %)	126
Yes	AIS	67 (93.06 %)	5 (6.94 %)	72
	**n**	**243**	**15**	**258**

AIS differs not from non-AIS; p = 0.4660 by Pearson

Analysis of the single factors together with the influence of treatment, i.e. using AIS catheters or not, on the risk of a shunt infection, showed that only a former shunt infection (see Table 2) were significantly related to a higher risk for suffering from shunt infection. The use of AIS or non-AIS had no significant advantage. (p = 0.8611; multiple logistic regression). Also significantly related to a shunt infection was the number of shunt surgeries. 598 surgical interventions had been performed, 190 interventions in the AIS group (2.21 interventions per patient), 408 in the non-AIS group (2.37 interventions per patient) (Wilcoxon test, p = 0.3063). There was no shunt infection in the group of patients on whom only one shunt surgery was performed. In patients who had undergone at least two shunt surgeries, we observed 15 out of 166 patients with shunt infections, i.e. 9% (p = 0.0015; Fisher's exact test). Comparing the 52 patients with AIS and the 114 patients without AIS with respect to the incidence of shunt infections, we obtain 5/52 (9.6 %) and 10/114 (8.8 %), respectively (p = 1.0; Fisher's exact test), see Table 6.

**Table 6:** Comparing rate of shuntinfection by the number of shunt surgery and AIS vs non-AIS

No. of shunt surgery		No shuntinfection	Shuntinfection	N
1	non-AIS	58 (100 %)	0 (0 %)	58
1	AIS	34 (100 %)	0 (0 %)	34
2 or more	non-AIS	104 (91.23 %)	10 (8.77 %)	114
2 or more	AIS	47 (90.38 %)	5 (9.62 %)	52
	**n**	**243**	**15**	**258**

AIS does not differ from non-AIS (p = 0.8611) but No. of shunt surgery >1 differs significantly from No. = 1 (p = 0.0002); (multiple logistic regression)

In Table 7, patients who suffered from shunt infection are presented in detail. Fourteen infections occurred within the first 6 months after shunt insertion independently of the type of shunt used (AIS or non-AIS). The most frequent pathogen for shunt infection was S. epidermidis. In the AIS group one out of five bacteria could not be classified, furthermore no antibiogram was available. Only in one patient the S. epidermidis was resistant to clindamycin and rifampicin. In the other three patients S. epidermidis and S. aureus were sensitive to both antibiotics. In the non-AIS shunt infection group, only two patients had an infection with S. aureus or S. epidermidis, which were sensitive to both antibiotics (clindamycin and rifampicin).

**Table 7:** Shunt infections, clinical data, AIS vs non-AIS, pathogens and antibiograms.

ID	Age [year]	Sex	No. of risk factors	Time to infection^1 ^[days]	AIS^2^	Organism	Rifampicin	Clindamycin
15	54.3	f	2	6	0	S. epidermidis	s	r
10	50.2	m	1	12	0	Citrobacter koseri	r	not tested
8	53.5	m	2	15	0	S. epidermidis	s	r
1	61.3	f	4	15	0	S. epidermidis	r	r
11	72.1	m	2	22	0	S. epidermidis	s	s
5	25.4	m	0	28	0	S. epidermidis	r	r
3	65.4	f	2	36	0	S. epidermidis	s	r
14	45.0	f	2	148	0	S. aureus	s	s
6	4.0	f	3	170	0	S. epidermidis	s	s
7	77.5	f	1	375	0	Proprioni bacterium acnes	not tested	s

4	74.3	f	3	4	1	S. epidermidis	r	r
12	66.7	f	1	13	1	gram positive coccus*^1^	unknown	unknown
16	0.3	f	4	26/5	1	S. epidermidis	s	s
9	53.4	f	4	178	1	S. aureus*^2^	s	s
17	3.5	f	2	106/294	1	S. epidermidis	s	s

^1 ^Time to infection between shunt implantation and shunt explantation.

^2 ^AIS: Antibiotic impregnated shunt: 0 = no, 1 = yes

*^1 ^gram positive coccus, no specific differentiation possible; *^2 ^Methicillin resistent

Patient ID 9 showed a skin ulceration 178 days after shunt implantation, patient ID 14 showed a galea ulceration after 148 days.

Patient ID 16. This prematurely born patient had her first shunt implantation at day 13 postpartum. Three months later the shunt system was changed due to dysfunction. 20 days later the child suffered from a liquor fistula, another surgery was done. 6 days later the first liquor infection with S. epidermidis was seen. The patient was treated with systemic antibiotics. Three months later another neurosurgical procedure was performed. A supratentorial cyst was opened by endoscopic surgery. Five days later the child had a second liquor infection with S. epidermidis, again the infection was treated by antibiotics.

Patient ID 17. This 3.5 year old child, who was born in the 25^th ^week of pregnancy, had her first shunt implantation after intraventricular hemorrhage. In the age of 3.5 years a shunt revision was necessary, because the supratentorial ventricles were collapsed and the fourth ventricle was enlarged. Three months later a second surgery was necessary because the catheter in the fourth ventricle had to be replaced. 12 days later the child suffered from shunt infection, the shunt was removed, liquor was temporarily drained by an external ventricular drainage. Additionally she got systemic antibiotic therapy. 18 days later a new AIS system was implanted. Unfortunately, the child suffered from external shunt infection ten months later, and the complete system had to be removed again.

In all patients no AIS-correlated complications occurred, specifically there were no toxic or allergic complications. No patient presented epileptic problems after antibiotics impregnated shunt implantation. Only one infection was cured merely by systemic antibiotics. In all other patients the complete shunt system was removed, followed by temporary external liquor drainage and systemic antibiotic treatment. There was no failure of infection therapy.

## Discussion

Shunt infections result mostly from intra-operative contamination by coagulase-negative staphylococci and other skin flora [20,21]. After adhesion to the implanted surface, the coagulase-negative staphylococci produce an extracellular mucoid biofilm, called ‚slime‘, embedding the bacteria and protecting them from the antibiotics [22-24]. Based on these findings, effective prophylaxis of infection begins with the prevention of initial bacterial adhesion to the shunt material. One way to establish this is to optimize the operating theatre and the shunt procedures as described by Choux et al. in 1992 [13] and Choksey and Malik in 2004 [11]. This may be possible, if at all, in hospitals with highly trained specialized neurosurgeons and nurses. In teaching hospitals with trainees and in developing countries it will be difficult to achieve these standards. Therefore, additional efforts for preventing shunt infections are necessary. One method is to reduce the risk factors causing infection. Reducing risk factors causing cerebrospinal fluid shunt infections is a controversial debate. In many reports risk factors like the different types of hydrocephalus [25,26], young age of the patient [27], presence of a previous external drainage or shunt system are accepted as unsusceptible risk factors. Susceptible risk factors for shunt infection are the duration of shunt surgery and the number of people present in the operating room [13].

In a prospective study 2001 Kulkarni et al. investigated risk factors causing shunt infections [28]. They found that only postoperative cerebrospinal fluid leakage, young age of the patients (premature infants), and perforated surgeon's gloves were significant risk factors for shunt infections. No common consensus exists at all for risk factors responsible for shunt infection.

In the presented data a significant correlation between developing shunt infection existed only for the number of surgeries and former shunt infection. The presented data show similar infection rates in the compared groups (AIS versus non-AIS). In the AIS group, in which 83.7 % of the patients had at least one risk factor, 5.81 % suffered from shunt infection. In the non-AIS group, in which 73.3 % had risk factors, 5.81 % had a shunt infection. Although some reader may suspect that some bias in the two groups may be possible, statistical analysis demonstrated no significant differences in both groups with only one exception, i.e. younger mean age of the AIS group.

In contrast to the risk factors, high consensus exists in the literature regarding the pathogens causing shunt infections. Coagulase-negative staphylococci, mostly derived from the patient's skin flora during surgery, colonize the inner surfaces of the shunt tubing [20,29,30]. Staphylococci (S. epidermidis and aureus) are the bacteria that cause shunt infections most frequently [31]. Gram negative bacteria were found less frequently. The presented data are in accordance with these findings. The most frequent bacteria noted was S. epidermidis. Remarkably, three out of six infections in the AIS group were caused by S. epidermidis or aureus, which were sensitive to clindamycin and rifampicin. It is assumed that the contamination of the shunts happened after shunt implantation. In only one case, the causing bacteria were resistant to both antibiotics.

More than 90 % of infections are diagnosed within the first 12 months from surgery [32]. In our study one patient had a late shunt infection, caused by propionibacterium acnes (375 days). Schiff et al. reported a rate of 1 % late shunt infections, defined as 6 months after surgery, the most common organisms were propionibacterium species and S. epidermidis species [33]. There are several causes for the failure of infection protection. According to Bayston et al., it takes 48 to 58 h to destroy 100 % of the adhered bacteria, the protective activity of the impregnated shunt has a duration of more than 50 days [34]. Recently Pattavilakom et al. reported about antimicrobial activity for a period of up to 127 days after ex vivo explantation of antibiotic impregnated cerebrospinal fluid catheters [35].

A possible explanation for the failure of infection prophylaxis in the AIS group could be the fact that the ventricular catheter and the distal shunt catheter are antibiotic-impregnated, whereas the valve and the Rickham reservoir are not.

Other possible explanations could be further invasive interventions such as neurosurgical procedures or skin ulcerations. In the presented data patient ID 9 showed ulceration of the skin. The ulceration in this severely disabled patient had its manifestation 178 days after implantation of the shunt. This explains the infection time. The shunt infection was obviously caused by the ulceration which cannot be prevented by AIS. In the non-AIS group, patient ID 14 was in a comparable situation. This patient had an ulceration of her galea. The shunt system had to be removed after 148 days. As there were late infections based on ulcerations of the galea in both groups, we included both patients in the statistical analysis, otherwise the total infection rate would be incorrectly too low.

Comparing AIS with non-AIS, the presented data could not demonstrate a significant reduction of shunt infection by AIS. Remarkably we found three out of five infections in the AIS group which were caused by skin ulcerations or neurosurgerical procedures highly susceptible for contamination after initial shunt implantation. Furthermore, the infection rate in the non-AIS group was obviously smaller than reported in the literature. Sciubba et al. [36] retrospectively investigated 211 pediatric patients with 353 shunt placement procedures over a 3-year period. In the first 18 months, 208 shunts were placed with non-impregnated catheters. 12 % of these patients had shunt infections compared to only two patients (1.4 %) out of 141 in whom AIS had been inserted in the second 18 months. The authors could demonstrate a significant decrease in the infection rate in the AIS group by changing their management. Aryan [2] investigated 32 Bactiseal shunt implantations in 31 children aged from 6 months to 17 years and compared them to patients treated with non-impregnated shunts. In the AIS group 12.5 % early and 18.8 % late complications occurred compared to 23.4 and 34.8 % in the non-AIS group.

Prospective randomized blind studies are highly appreciated. To show a statistically significant decrease from 6 to 3 % shunt infections, a prospective study with 1628 patients would be necessary presuming a significance level of 5 % using by Fisher's exact test. To the best knowledge of the authors only prospective studies with a fewer number of patients exist. Govender and coworkers [37] showed a significantly decreased infection rate from 16 % in the control group to 6 % in the AIS group in a prospective randomized blind study including 110 patients. Our infection rate in the AIS group is comparable to their results. In contrast to Govender et al. and Sciubba et al. [36], the risk of shunt infection in non-AIS patients in this study was very low (5.81 %). The study of Govender et al. was performed in a single center in South Africa, where diverse problems such as a high infection rate with HIV and malnutrition compared to highly industrialized countries exist. This might explain the high infection rate in their non-AIS group.

The infection rate in our investigation did not achieve the excellent results reported by Choux et al. and Chouksey et al. [11,13]. In the cited reports, shunt operations were performed by one very experienced surgeon with the aim to reduce shunt infections. In the actual investigation surgery was done by various surgeons, including residents in neurosurgery with an infection rate of 6 % in all patients. This is probably general practice in most neurosurgical training centers at university hospitals.

AIS did not reduce the infection rate in the presented study. Nevertheless it must be noted that in the AIS group three patients suffered from shunt infections caused by skin ulceration or neurosurgical procedures with exposition of the cerebrospinal liquor after shunt implantation. AIS was not developed to prevent infection in such cases, therefore an advantage of AIS is not excluded.

## Conclusion

Using AIS or not it is still important to obey consequently the surgical principles of aseptic and atraumatical preparation. Based on the presented data and the small number of reported studies a prospective randomized multicenter study is required.

## List of abbreviations

AIS antibiotic-impregnated shunts

Non-AIS non antibiotic-impregnated shunts

CSF cerebrospinal fluid

OR odds ratio

CI confidence interval

## Competing interests

The authors declare that they have no competing interests.

## Authors' contributions

Rainer Ritz Acquisition of data, analysis and interpretation of data, drafting the manuscript

Florian Roser Acquistion of data, revising the manuscript critically

Matthias Morgalla Involved in drafting and translation of the manuscript

Klaus Dietz Statistical analysis

Marcos Tatagiba Revising the manuscript critically for important intellectual content

Bernd E. Will Conception and design of the study

All authors read and approved the final manuscript.

## Pre-publication history

The pre-publication history for this paper can be accessed here:


